# Therapeutic Target Discovery Using High-Throughput Genetic Screens in Acute Myeloid Leukemia

**DOI:** 10.3390/cells9081888

**Published:** 2020-08-12

**Authors:** Qiao Liu, Michelle Garcia, Shaoyuan Wang, Chun-Wei Chen

**Affiliations:** 1 Fujian Provincial Key Laboratory on Hematology, Department of Hematology, Fujian Institute of Hematology, Fujian Medical University Union Hospital, Fuzhou 350108, China; qialiu@coh.org (Q.L.); shaoyuanwang@mail.fjmu.edu.cn (S.W.); 2 Union Clinical Medical College, Fujian Medical University, Fuzhou 350108, China; 3 Department of Systems Biology, Beckman Research Institute of City of Hope, Duarte, CA 91010, USA; mgab2018@mymail.pomona.edu; 4 Pomona College, Claremont, CA 91711, USA

**Keywords:** high-throughput, genetic screen, CRISPR, shRNA, genome-wide, epigenetics, drug resistance, AML, leukemia

## Abstract

The development of high-throughput gene manipulating tools such as short hairpin RNA (shRNA) and CRISPR/Cas9 libraries has enabled robust characterization of novel functional genes contributing to the pathological states of the diseases. In acute myeloid leukemia (AML), these genetic screen approaches have been used to identify effector genes with previously unknown roles in AML. These AML-related genes centralize alongside the cellular pathways mediating epigenetics, signaling transduction, transcriptional regulation, and energy metabolism. The shRNA/CRISPR genetic screens also realized an array of candidate genes amenable to pharmaceutical targeting. This review aims to summarize genes, mechanisms, and potential therapeutic strategies found via high-throughput genetic screens in AML. We also discuss the potential of these findings to instruct novel AML therapies for combating drug resistance in this genetically heterogeneous disease.

## 1. Introduction

Acute myeloid leukemia (AML) is one of the most aggressive forms of hematopoietic disorders. Estimated by the American Cancer Society, there will be about 20,000 new cases of AML and nearly 12,000 deaths from AML in the United States for 2020. Even with intensive chemotherapy and allogeneic hematopoietic stem cell transplantation, the survival outcomes of AML patients remain remarkably low [1]. The heterogeneity of mutations and the drug-resistant potential of leukemic stem cells (LSCs) in AML patients lead to a profound relapse frequency of this disease after conventional treatment [2]. Nevertheless, with increased exploration of AML biology in recent years, therapeutic strategies have been revolutionized by combining chemotherapies with small-molecule inhibitors that target additional AML-driven genes [3]. Moreover, a detailed evaluation of the genetic background in AML patients via next-generational sequencing enables a more accurate diagnosis and personalized therapeutic strategy [4].

**Figure 1:**
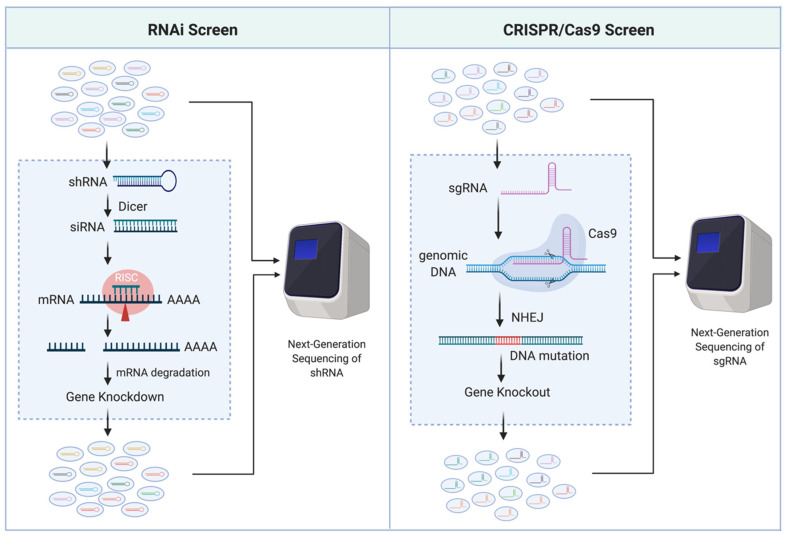
Scheme of RNAi and CRISPR/Cas9 high-throughput functional genetic screens.

Traditionally, uncovering AML genotype-to-phenotype relationships has been heavily reliant on sequencing clinical samples, identifying AML-associated mutations, and subsequently mutating and/or altering gene expression levels in a laboratory setting to observe a phenotype of interest. Impressively, the number of genes identified as related to AML survival has drastically increased in the past decade, primarily credited to the availability of novel genetic screening technologies such as RNA interference (RNAi) [5,6,7] and clustered regularly interspaced short palindromic repeats (CRISPR)/Cas9 [8,9]. RNAi is a post-transcriptional gene silencing (i.e., knockdown) mechanism. It utilizes double-stranded RNA, such as short hairpin RNA (shRNA), that can be processed by Dicer (an endoribonuclease) to produce small interfering RNA (siRNA; 20–25 nucleotide) fragments and incorporated into the RNA-induced silencing complex (RISC) to degrade the sequence complementary mRNA. CRISPR/Cas9, on the other hand, is a gene-editing (i.e., knockout) system that leads to disruption of the gene coding sequences. It utilizes single-guide RNA (sgRNA) combined with the Cas9 endonuclease to induce double-strand breaks of the guide (17–20 nucleotide) sequence matched DNA locus, resulting in random mutations through the error-prone non-homologous end-joining (NHEJ) DNA repairs. The optimization of these sequence-specific gene-modulation systems combined with next-generation sequencing has made these tools popular for high-throughput functional genetic screening (Figure 1).

Since 2006, several research groups have been dedicated to providing genome-wide and pathway-focused libraries for functional genetic screens. For example, The RNAi Consortium (TRC) shRNA library [10] and the genome-scale CRISPR-Cas9 knockout (GeCKO) sgRNA library [11] are two popular genome-wide library consortiums targeting more than 10,000 genes. The development of computational algorithms (e.g., MAGeCK) enables the prioritization of candidate genes from genome-scale knockout screens for further validation [12]. These high-throughput genetic screen/analysis tools provide the advantage of vigorously finding functionally essential genes in an unbiased manner. The data emerging from this relatively new approach have discovered AML-related mechanisms that contribute to a more in-depth understanding of AML etiology and highlight a unique array of potential therapeutic options (Table 1).

## 2. Epigenetic Regulators

The genome in eukaryotic cells is orchestrated in the form of the chromatin fiber, which has a basic unit, the nucleosome, that consists of four types of histone proteins (H2A, H2B, H3, and H4) surrounded by ~150 base pairs of DNA [39]. Chromatin has two cytologically visible ground states that include active and repressed genomic regions, named euchromatin and heterochromatin, respectively. The interchange between chromatin states occurs via protein-mediated modifications on DNA and histones, which plays a central role in transcriptional regulation and cell-fate determination [40]. The dynamic interplay between such modifications and their cofactors embodies “epigenetics,” i.e., a heritable form of information that modifies gene expression without altering the sequence of underlying DNA.

Epigenetic cofactors include enzymes that modify nucleotide bases and histone tails (writers), proteins that recognize these modifications by binding to specific epigenetic alterations (readers), and enzymes that remove the epigenetic modifications (erasers) [41]. The essential roles of these epigenetic regulators in oncogenic gene expression have been studied intensively and have attracted research efforts in both basic science and pharmaceutical industry as a field for identifying new arrays of potential therapeutic targets [42,43].

### 2.1. Histone Writers

Histone writers such as lysine methyltransferases (KMTs) and lysine acetyltransferases (KATs) are a subclass of epigenetic writers that modify histones through the addition of methyl and acetyl moieties [40]. KMTs methylate lysine residues, resulting in three possible modification states: mono-, di-, or trimethylation. In particular, methylation on lysine residues 9 and 27 of histone H3 (H3K9, H3K27) is highly associated with transcriptional repression, whereas methylation on H3K4 and H3K79 is relevant to transcriptional initiation [44]. For example, the disruptor of telomeric silencing 1-like (DOT1L) protein specifically mediates methylation of H3K79 and induces expression of downstream genes including homeobox A (HOXA) clusters (required for embryonic development) and MEIS1 (required for hematopoiesis) in MLL-r leukemia—an aggressive family of acute leukemia driven by rearrangement of the mixed lineage leukemia (MLL1) gene [45,46]. KATs acetylate histones at various positions (H3K9, H3K14, H3K27, H4K12, and H4K16, etc.), and these modifications are generally associated with an open, accessible chromatin state [47]. Several KATs such as KAT3A (CBP) and KAT3B (p300) are known to be required for AML cell survival [48,49]. Here, we summarize the histone writers found specifically through genetic library screens in AML.

#### 2.1.1. Males Absent on the First (MOF)

**Figure 2:**
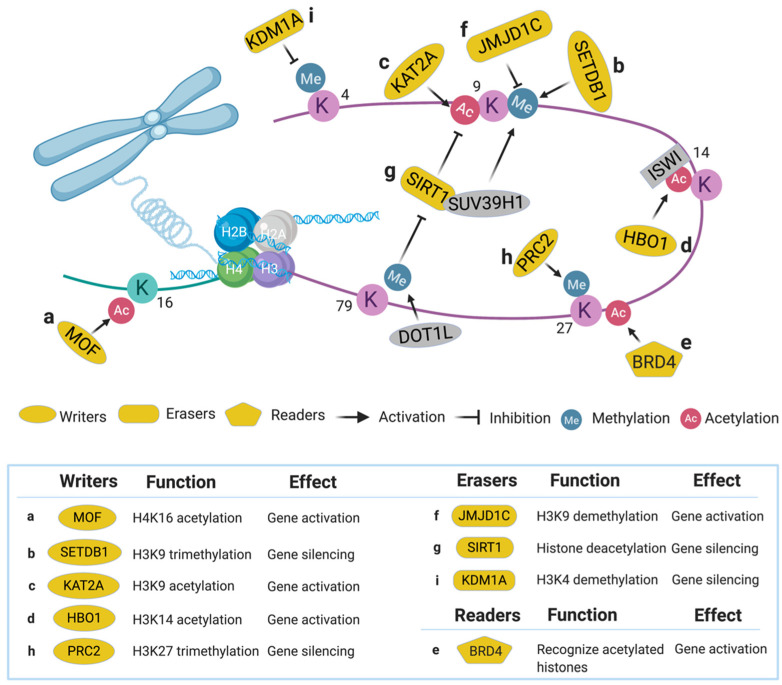
Epigenetic regulators identified via high-throughput genetic screens in acute myeloid leukemia (AML).

MOF, also called KAT8, is a member of the MYST acetyltransferase family that is characterized by the presence of the highly conserved acetyl-CoA binding motif (MYST domain) [50] and is involved in adult hematopoiesis through acetylation at H4K16 [51] (Figure 2a). MOF was identified in an shRNA library screen (2252 shRNAs targeting 468 chromatin regulators) aimed at finding novel druggable targets in the epigenetic pathways that maintain AML driven by MLL-AF9, a type of MLL-r characterized by the translocation of MLL1 and AF9 genes [13]. Twenty shRNAs in the screen were strongly depleted (indicating functionally essential for cell survival), whereas MOF is one of the only two genes that had more than one shRNA identified in this category. In vivo experiments revealed that suppression of MOF causes decreased leukemogenesis through induction of DNA damage, indicating MOF may serve as a potential therapeutic target in MLL-r leukemia for further pharmacological development [52].

#### 2.1.2. SET Domain Bifurcated 1 (SETDB1)

SETDB1, a SET domain-containing KMT, is a transcriptional repressor via trimethylation of H3K9 (Figure 2b) [53,54]. Recently, SETDB1 was identified as critical for AML cell survival in a CRISPR/Cas9 screen [14]. In this study, sgRNAs targeting approximately 350 human epigenetic and transcriptional modifiers (15–25 sgRNAs per gene) were transduced into the human AML cell line THP-1, revealing that SETDB1 was one of the top essential genes in AML. Suppression of SETDB1 in AML promotes the expression of endogenous retroviruses and long interspersed nuclear elements, thereby triggering the RNA-sensing pathways and interferon-induced apoptosis through increased dsRNA content in the cells [55]. Since aberrant expression of SETDB1 was found in various human cancers, inhibitors such as DZNep were developed for targeting this pathway [56].

#### 2.1.3. Lysine Acetyltransferase 2A (KAT2A)

KAT2A, also known as general control nonderepressible-5 (GCN5), is an enzyme involved in acetylation of H3K9 (Figure 2c) and succinylation of H3K79 [57,58]. In a genome-scale sgRNA library screen (90,709 sgRNAs targeting 18,010 genes) aimed at identifying genetic vulnerabilities in AML, KAT2A was identified as an essential gene in a sub-set of AML models [15]. Pharmacological inhibition of KAT2A by Butyrolactone 3 (MB-3) led to downregulation of leukemogenic genes such as HOXA9 and MEIS1, and upregulation of myeloid differentiation cell surface proteins including CD13, CD18, and CD11b [15]. These results indicate KAT2A could be a potential therapeutic target in AML.

#### 2.1.4. Histone Acetyltransferase Binding to ORC1 (HBO1)

HBO1, an MYST acetyltransferase also known as KAT7, specifically acetylates H3K14 (Figure 2d) to regulate gene transcription [59] and embryonic development [60]. In an MLL-AF9 AML cell survival screen using 1922 shRNAs targeting 270 chromatin modifiers, HBO1 was chosen for further study due to its novel correlation with LSC maintenance [16]. Suppression of HBO1 led to apoptosis, differentiation, and cell cycle arrest in LSC. Using a CRISPR domain screen approach, the authors confirmed the histone acetyltransferase domain of HBO1 is essential to the acetylation of H3K14. This histone modification can be recognized by ISWI family chromatin remodeling proteins, which facilitate the processivity of RNA polymerase II to maintain the high expression of leukemic genes, thereby, sustaining the functional properties of LSC. These results demonstrate the substantial roles of HBO1 in maintaining LSC; thus, it is a plausible therapeutic target for AML.

### 2.2. Histone Readers

The epigenetic modifications deposited by writers on chromatin are recognized by nuclear proteins with specific binding domains, known as readers. For example, Bromodomains and YEATS domains recognize multiple types of acetylated histones [41]. On the other hand, PHD fingers and chromodomains bind to methylated lysine(s)/arginine(s) on histones [41]. These epigenetic readers play a central role in establishing the identities between active euchromatin and repressed heterochromatin, and this represents an attractive emerging field for pharmaceutical targeting.

#### 2.2.1. Bromodomain-Containing Protein 4 (BRD4)

BRD4, a member of the bromodomain and extra terminal domain (BET) family, recognizes several acetylated positions on H3 and H4 (Figure 2e) [61]. BRD4 was identified as a top-hit in an epigenetic-focused shRNA library screen (1094 shRNAs targeting 243 chromatin regulators) aimed at identifying genes required for AML maintenance [17]. BRD4 maintains AML survival by binding to the promoter and enhancer of MYC, a known oncogenic driver gene [62]. Suppressing BRD4 by shRNAs or a bromodomain inhibitor JQ1 showed significant anti-leukemic effects in vitro and in vivo. Results from this study demonstrated the utility of bromodomain/BET small-molecule inhibitors in AML treatment [17,63] and casted a new field of targeting histone reader domains for cancer therapy [64,65,66].

#### 2.2.2. Eleven-Nineteen Leukemia (ENL)

ENL, a YEATS domain-containing protein, is one of the MLL translocation partner genes frequently observed in MLL-r leukemia patients [67,68]. It is also one of the core components of the super elongation complex (SEC) that regulates the transcriptional elongation of RNA polymerase II [69]. To illuminate new genetic dependencies in MLL-r leukemia, a genome-scale CRISPR/Cas9 knockout screen was performed in MLL-r leukemia cells [18]. This study revealed that ENL is highly essential to the proliferation of MLL-r leukemia, and depletion of ENL inhibits the initiation and elongation by RNA polymerase II and suppresses MLL-r targeted gene transcription. Since the YEATS domains of ENL [70] and AF9 [62] are responsible for recognizing lysine acylation and have been demonstrated essential in AML, inhibitors were developed targeting this chromatin module [71] for further investigation.

### 2.3. Histone Erasers

The methyl- and acetyl-modifications made by histone writers can be removed by specific enzymes known as histone erasers. Demethylation of lysine residues relies on two different families: the lysine-specific demethylase (LSD) family and the Jumonji C (JmjC) domain family [72]. The LSD family, including KDM1A (LSD1) and KDM1B (LSD2), is characterized by a C-terminal amine oxidase domain that is functionally important for demethylation. The JmjC family, such as KDM2–8, removes methylation via dioxygenase activity [73]. In this category, KDM2B (also known as JHDM1B) promotes AML cell proliferation by regulating the cell cycle [74]. Deacetylation of lysine residues mainly occurs through two protein deacetylase families, i.e., the histone deacetylase family (HDACs) and NAD-dependent protein deacetylase Sirtuin family (SIRTs) [75,76]. HDAC1 maintains AML survival by inhibiting Kruppel-like factor 4 (Klf4) expression [77], whereas HDAC8 inactivates p53 and promotes AML proliferation [78]. These studies highlight an essential role of histone erasers in AML biology.

#### 2.3.1. Jumonji Domain Containing 1C (JMJD1C)

JMJD1C, a predicted histone demethylase at H3K9 (Figure 2f), was a top hit in two shRNA library screens in MLL-AF9 leukemia [19,79]. In one in vivo shRNA screen, a custom pool of 752 shRNAs that targets 160 genes identified from a ChIP-seq of MLL-AF9 bound loci in AML was developed [19]. The AML cells transduced with this library were transplanted into sub-lethally irradiated recipient mice. Upon development of AML, the bone marrow and spleen of the recipient mice were collected for high-throughput sequencing. In this study, JMJD1C was one of the top hits along with other well-characterized leukemia driver genes, including HOXA9 and HOXA10. Conditional knockout of Jmjd1c in mice suggested an essential role for JMJD1C in maintaining LSCs in AML. JMJD1C was further demonstrated to regulate the HOXA9 gene-expression program via direct interaction with HOXA9 [19]. Another in vitro shRNA library screen targeting epigenetic factors (898 constructs targeting 319 chromatin regulators) also found JMJD1C to be the highest scoring hit in MLL-AF9 leukemia [79]. In this study, depletion of JMJD1C led to deregulation of MYC, MYB, and HOXA9/MEIS1 target genes. Recently, JMJD1C inhibitors have been developed to induce preferential death of MLL-r AML cells, suggesting that targeting JMJD1C could be a promising therapeutic strategy [80].

#### 2.3.2. Sirtuin 1 (SIRT1)

SIRT1, a NAD-dependent protein deacetylase, can deacetylate multiple proteins, including histones, FOXO1, NFκB, etc. [81,82]. In chronic myelogenous leukemia, SIRT1 inhibits tumor suppressor gene p53 (TP53) through deacetylation and contributes to LSC maintenance [83]. In a study aimed at identifying effectors mediating DOT1L inhibitory therapy in MLL-r AML, a genome-wide shRNA screen (92,425 shRNAs targeting 16,924 mouse genes) was performed in mouse Dot1l^f/f^-MLL-AF9 leukemic cells [20]. This study discovered that SIRT1-mediated histone deacetylation is required for silencing the MLL-r target genes after DOT1L inhibition. SIRT1 also promotes H3K9 methylation through recruiting a KMT called SUV39H1 (Figure 2g), resulting in a heterochromatin-like state around the MLL-r targeted loci [20]. These results underline the dynamic interplay between chromatin regulators and could provide novel opportunities for combination therapies.

## 3. Kinase Pathways

Kinases are a class of ATP-dependent enzymes that play critical roles in cellular signaling by phosphorylating selective downstream substrates. The abnormal expressions of kinases such as cyclin-dependent kinases (CDKs) [84] and mitogen-activated protein kinases (MAPKs) [85] are involved in AML cell proliferation, and a vast array of inhibitors targeting kinases have been developed [86]. For example, the treatment of FLT3 inhibitors (sunitinib and midostaurin) has demonstrated an overall improvement in AML patients with FLT3 mutations [87]. With the advance of genetic screen technologies, an increased number of kinases have now been identified as potential therapeutic targets in AML.

### 3.1. Glycogen Synthase Kinase 3 (GSK3)

GSK3, a serine/threonine-protein kinase, is involved in cellular proliferation, migration, and apoptosis [86]. It was identified in a study aimed at identifying novel druggable kinases in AML, in which the authors integrated a kinome-focused shRNA library screen (~5000 shRNAs targeting ~1000 kinase genes) with a kinase inhibitor-focused small-molecule library screen (84 compounds) [21]. In this study, targeting GSK3 emerged as a top candidate for eradicating AML, as both chemical and shRNA-mediated inhibition of GSK3 led to the differentiation of AML cells via downregulation of MYC expression. GSK3 was also reported as a target of MLL-r leukemia that fosters cell proliferation by promoting HOX-associated transcription [88]. Several GSK3 inhibitors have been developed, and some have undergone clinical trials [89], supporting the potential of targeting GSK3 for AML therapy.

### 3.2. Rho-Associated Protein Kinase 1 (ROCK1)

**Figure 3:**
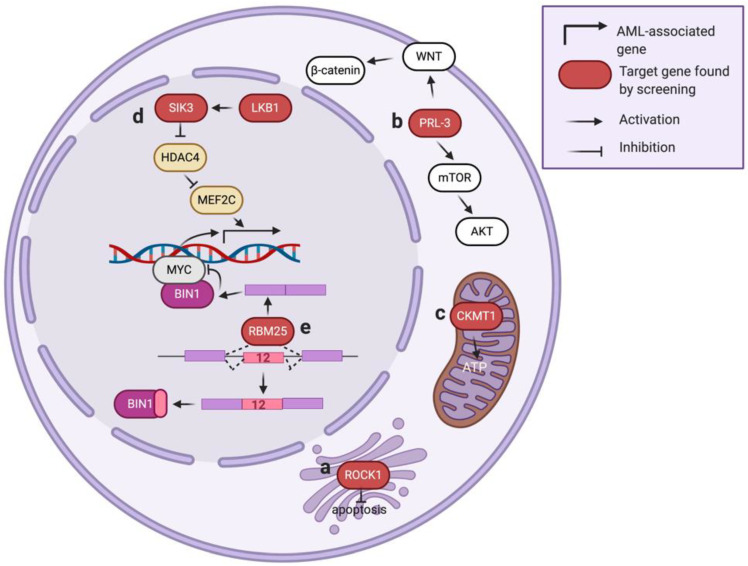
Kinases and gene expression-related genes identified via high-throughput genetic screens in AML.

ROCK1, also known as ROKβ, is a serine/threonine-protein kinase located on the Golgi apparatus and plays a crucial role in regulating cell motility and angiogenesis (Figure 3a) [90,91]. Several studies imply ROCK1 is also involved in mitochondrial injury response and cellular apoptosis [92,93]. In a loss-of-function RNAi library screen (7709 shRNAs with ~5 shRNAs per target gene) in primary leukemic cells, ROCK1 was found as one of the top essential genes in AML [22]. Suppression of ROCK1 resulted in increased apoptosis and decreased viability of primary AML cells. The authors further demonstrated the potential of ROCK1 inhibitor fasudil in AML therapy using a xenograft model, delineating a potential therapeutic strategy in AML patients.

### 3.3. Phosphatase of Regenerating Liver 3 (PRL-3)

Increased PRL-3 (encoded by protein tyrosine phosphatase type IVA 3, or PRP4A3) expression is associated with AML development [94,95]. However, pharmacological targeting of PRL-3 remains a challenge due to the relatively shallow pocket of the catalytic site in PRL-3 [96]. In one study aimed at finding druggable gene candidates for AML with high PRL-3, a whole-genome RNAi library screen (80,000 shRNA targeting 16,000 genes) was performed in AML cells co-transduced with an RPL-3 overexpression construct [23]. This study identified a strong association of mTOR/AKT and WNT/β-catenin signaling pathways in the PRL-3-high AML (Figure 3b). As both of these signaling cascades play essential roles in tumorigenesis [97,98], further experiments demonstrated the combination of an mTOR/AKT inhibitor (VS-5584) and a WNT/β-catenin inhibitor (IGC-001) leads to a synergistic killing effect selectively in AML with high PRL-3 expression. These results suggest that high-throughput genetic screens can discover cooperative signaling networks and facilitate the development of novel therapeutic strategies.

### 3.4. Creatine Kinase, Mitochondrial 1 (CKMT1)

CKMT1 is a creatine kinase located in mitochondria that catalyzes the bi-directional conversion between creatine/ATP and phosphocreatine/ADP (Figure 3c), thereby modulating the buffering and regeneration of ATP in the high-energy-demanding cells. In a metabolism-focused library screen (361 shRNA targeting 67 genes in glycolysis and TCA cycle pathways) [24], CKMT1 was identified as necessary for the survival of AML with a high level of EVI1, a proto-oncogene whose expression level is associated with adverse prognosis in AML patients [99]. This study identified that EVI1 could induce the expression of CKMT1, thus promoting mitochondrial function and ATP production in the EVI1-positive AML. Results from this study suggest that targeting CKMT1 or creatine metabolism may provide a promising therapeutic strategy for EVI1-driven AML that is highly resistant to conventional treatment.

### 3.5. Liver Kinase B1 (LKB1)

LKB1 is a serine/threonine-protein kinase that regulates cell processes such as DNA damage response, cell polarity, and apoptosis [100,101]. In a kinase-focused sgRNA library screen (targeting 482 protein kinases with ~6 sgRNA per gene focused on the kinase domains of each candidate gene) of twenty-six cell line models (including eight AML cell lines), LKB1 was identified as a selectively essential gene in AML [25]. The authors defined that LBK1 phosphorylates its downstream effector SIK3 [102], leading to further phosphorylation and suppression of histone deacetylase HDAC4. This mechanism allows maintenance of the histone H3K27 acetylation (associate with gene expression) at the enhancer loci targeted by MEF2C, a leukemic transcription factor in AML (Figure 3d) [103]. Targeting LKB1 or SIK3 decreases histone acetylation at MEF2C-bound enhancers and suppresses AML proliferation, highlighting a novel strategy of disabling an oncogenic transcription factor by targeting its upstream kinases.

## 4. Gene Expression Regulators

Gene expression prior to protein translation can be divided into two major steps: gene transcription and RNA processing. Dysregulation of these steps may mislead the expression of cancer-related genes and trigger leukemia development [104]. For example, mutations of transcription factor RUNX1 support AML development by promoting the expression of hematopoiesis and cell cycle-related genes [105]. Mutations in the RNA splicing factors SF3B1 and SRSF2 are frequently found in AML patients and are associated with drug resistance [106]. These studies underscore a strong relationship between gene expression regulators and AML pathogenesis. Here, we summarize AML-related gene expression regulators identified from functional genetic screens.

### 4.1. Zinc Finger E-Box-Binding Homeobox2 (ZEB2)

ZEB2, also known as SIP1, is associated with early fetal development through the TGFβ pathway and is known to promote epithelial–mesenchymal transition (EMT) by repressing the expression of epithelial cell–cell junction genes [107]. In a genome-scale shRNA screen project (17 AML cell lines compared to ~200 non-AML cell lines), ZEB2 was identified as a novel dependency in AML [26]. Depletion of ZEB2 impairs cell proliferation and induces differentiation in AML cells. Mechanistically, ZEB2 supports AML via recruiting of CtBP transcriptional repressor complexes to block myeloid differentiation. Results from this study extend the role of ZEB2 beyond regulating EMT and establish ZEB2 as a novel regulator of AML proliferation and differentiation.

### 4.2. Zinc Finger Protein 64 (ZFP64)

ZFP64, also called ZNF338, interacts with the transmembrane receptor Notch1 in myoblast cells [108] and is associated with the transcription factor NF-kB in macrophages [109]. ZFP64 was shown as required for MLL-r leukemia survival in a project of domain-focused CRISPR/Cas9 screens that target 1426 DNA-binding domains (total 8658 sgRNAs) over 33 cancer cell lines [27]. The authors discovered the specificity of ZPF64 for MLL-r leukemia is accounted for by an excessive density of ZFP64 binding motifs within the MLL gene promoter. Results from this study inspire a new vision into the transcriptional addiction in cancer based on the sequence anomaly of an oncogene promoter.

### 4.3. RNA-Binding Protein 25 (RBM25)

RBM25, an RNA-binding protein (RBP) that regulates pre-mRNA splicing, is known to mediate cell apoptosis by controlling splicing of the BCL2 family protein BCL2L1 [110]. To explore the role of RNA splicing in AML biology, an shRNA library targeting 230 splicing factors was screened in an in vivo AML mouse model [28]. In this study, RBM25 was identified as one of the top candidates that, when suppressed, can increase the fitness of AML in the transplanted mice. RBM25 suppresses AML progression via controlling the pre-mRNA splicing of BIN1, an endogenous inhibitor of MYC. Specifically, depletion of RBM25 leads to an aberrant expression of an inactive BIN1 isoform (contains exon 12) and results in an increased MYC activity (Figure 3e). A low RBM25 expression level is associated with high MYC activity and poor outcome in AML patients [28], indicating a tumor suppressor role of RBM25 in AML.

### 4.4. RNA-Binding Protein 39 (RBM39)

RBM39, also known as RNPC2, is another RBP identified from a library screening of 2900 sgRNAs targeting 490 RNA-binding domains of RBPs in multiple cancer cell models [29]. This study revealed that AML selectively requires RBM39, and loss of RBM39 leads to the destruction of an RBP interaction network and induces mRNA degradation resulting in AML cell death. Specifically, genetic or pharmacologic targeting of RBM39 promotes intron retention and represses cassette exon inclusion [111] prominently in HOXA9-regulated genes, of which many are required for AML development. This study also observed a profound sensitivity of spliceosomal mutant AML to RBM39 inhibition, providing an alternative strategy for the treatment of AML bearing RBP splicing mutations.

## 5. Therapeutic Response Modulators

The low overall survival rates observed in patients with AML reflect the enormous clinical and molecular heterogeneity of AML [1]. In addition to traditional chemotherapy (e.g., Cytarabine), the use of small-molecule inhibitors targeting leukemia-essential genes has become a popular strategy for AML treatment [112]. For instance, the FLT3 inhibitor sorafenib has undergone phase III clinical trials and is approved for the treatment of FLT3-ITD-positive AML [113]. Nevertheless, many of the targeted therapies are not sufficiently potent as a single regimen, and drug resistance has been reported [114]. Further research on drug-refractory mechanisms and combination with new molecular targeting may provide alternative options for AML patients. Here, we summarize the results of screens that identified genes involved in the AML therapeutic response.

### 5.1. Cytarabine

Cytarabine, also known as cytosine arabinoside (ara-C), is one of the most effective drugs for AML treatment over the past 40 years [115]. It kills proliferating cells by inhibiting DNA synthesis and blocking the G1-to-S phase transition. Despite the use of high-dose cytarabine in AML patients, the overall survival rate for AML patients remains lower than 30%. The AML cells that escape from cytarabine-induced G1/S phase blockade underscore a need to identify cytarabine resistance mechanisms and develop novel therapeutic strategies for AML treatment.

#### 5.1.1. Western Equine Encephalitis 1 (WEE1)

WEE1 is a nuclear-localized Ser/Thr-protein kinase involved in the transition between DNA replication and mitosis (S-to-G2 phase transition) through mediating CDK2 activation [116]. WEE1 was identified to offer cytarabine-resistance in AML by two independent studies published in 2012, one utilized a genome-wide shRNA library [117] and the other one used a kinase-focused shRNA library (targeting 572 kinases) [30]. Both studies observed that suppression of WEE1 by a small molecular kinase inhibitor MK1775 (AZD1175) [30,117] increased the sensitivity of AML cells to cytarabine through eliminating the CDK2 activity. These results emphasize the potential of a cytarabine/WEE1-inhibitor combination for advanced AML therapy.

#### 5.1.2. Deoxycytidine Kinase (DCK)

To understand pathways involved in chemo-resistance, a genome-wide CRISPR/Cas9 screen was conducted in AML cells treating with cytarabine [31]. In this study, disruption of DCK, a kinase participant in cytarabine phosphorylation to produce the biologically active ara-CTP, was identified to render AML cells resistant to cytarabine. The authors further conducted a screen of 446 US Food and Drug Administration (FDA) approved drugs and revealed that cytarabine-resistant cells exhibited increased sensitivity to prednisolone, suggesting an adjuvant therapy in chemo-refractory AML.

### 5.2. FLT3 Inhibitors

The FMS-like tyrosine kinase 3 (FLT3) gene is mutated in approximately 30% of AML patients [118]. Since patients with FLT3 mutations generally associate with a high risk of relapse and poor prognosis, multiple FLT3 inhibitors have been approved for clinical use in combination with the standard chemotherapies [119], while more FLT3 targeting compounds are in various stages of clinical development [3,87]. The profound impact of FLT3 inhibitors on AML patients depicts the need to understand the underlying mechanisms and perhaps develop additional combinational approaches to improve the efficacy of FLT3-targeted therapy in patients.

#### 5.2.1. Protein Sprouty Homology 3 (SPRY3)

SPRY3, an antagonist of the fibroblast growth factor (FGF) pathway [120], was identified as a therapeutic modulator in a genome-wide CRISPR library screen (64,751 sgRNAs targeting 18,080 genes) performed in an FLT3-mutant AML cell line (MV4-11) treated with a second-generation FLT-3 inhibitor AC220 [32]. The authors discovered that knockdown of SPRY3 triggers resistance to AC220 by reactivation of the FGF/Ras/ERK signaling pathway. This study also revealed that GSK3, a WNT signaling antagonist [121], is upstream to SPRY3 in AML. Importantly, inhibition of FGF (by PD161570), ERK (by U0126), or WNT (by PNU74654) signaling resensitizes the FLT3-mutant AML cells to AC220 treatment, providing a new option of targeting alternative signaling pathways in parallel to enhance the therapeutic response in AML.

#### 5.2.2. Ataxia Telangiectasia Mutated (ATM)

ATM is a serine/threonine kinase involved in activating the DNA damage checkpoint [122] and promoting the antioxidant response through activation of glucose-6-phosphate dehydrogenase (G6PD) [123]. To explore gene repression that sensitized AML to FLT3 inhibitors, an shRNA genome-wide library screen was performed in an FLT3-mutant AML cell line (Molm13) treated with the FLT3 inhibitor CEP-701 [33]. In this study, ATM was identified as a top candidate that, when depleted, will facilitate the cytotoxicity induced by FLT3 inhibition. Treatment of ATM inhibitor KU55933 led to higher AML sensitivity to CEP-701 through increased cell cycle arrest and apoptosis. Interestingly, the authored did not observe an increased DNA damage level; instead, they revealed the involvement of G6PD activity and cellular redox metabolism in the cells treated with this combinational regimen. These results argue the aberrant cellular metabolisms in the FLT3-mutant cases could offer a new direction of finding vulnerabilities in AML.

#### 5.2.3. Glutaminase (GLS)

GLS, the first enzyme catalyzing glutamine to glutamate to enter the TCA cycle [124], was identified from a genome-wide CRISPR screening aimed at identifying candidate targets that can synergize with FLT3-targeted therapy in AML [34]. The authors found that targeting GLS (by genetic ablation or an inhibitor CB839) induces synthetic lethality with AC220, specifically in the FLT3-mutant AML. Effects of combined FLT3 and GLS inhibition can be rescued by adding the glutamine downstream product α-ketoglutarate, suggesting a reprogramming of glutamine metabolism in this context. Mechanistically, glutaminase was shown to support the TCA cycle and glutathione synthesis following FLT3 inhibition. These results highlight glutamine metabolism, through its ability to support both mitochondrial function and cellular redox status, becomes a metabolic dependency of FLT3-mutant AML that can be combined with FLT3-inhibitor treatment.

### 5.3. Venetoclax

Venetoclax (also known as ABT-199) is an orally bioavailable small-molecule inhibitor that selectively targets BCL-2, a protein involved in antagonizing cellular apoptosis [125]. In 2016, the US Food and Drug Administration (FDA) approved venetoclax for the second-line treatment of patients with chronic lymphocytic leukemia with 17p deletion. In 2018, the FDA further approved venetoclax in combination with standard chemotherapy for AML patients who do not qualify for intensive therapy [126]. While the safety and overall response rate of the venetoclax therapy are favorable, the acquisition of resistance to venetoclax is the leading cause of treatment failure in these AML patients. It is crucial to explore the potential drug-resistant mechanisms and establish additional therapeutic regimens to benefit more AML patients.

#### 5.3.1. Tumor Protein 53 (TP53)

Mutation of tumor suppressor TP53 (also known as p53) is frequently observed in AML patients (5–20%) [127]. To understand the mechanisms underlying resistance to venetoclax in AML, one recent study utilized a genome-wide CRISPR/Cas9 screen (targeting 18,010 genes with ~5 sgRNA per gene) and identified the apoptotic network triggered by TP53 is required for the therapeutic response to BCL2 inhibition in AML [35]. Ablation of TP53 in AML cells renders resistance to venetoclax through disrupting the cellular apoptosis, mitochondrial homeostasis, and cellular metabolism. Importantly, the authors discovered that loss-of-function in TP53 induces aberrant activation of TRKs, a family of neurotrophin receptor tyrosine kinases. The increased sensitivity of TP53-deficient AML to the TRK inhibitors indicates a novel strategy to overcome venetoclax resistance in patients.

#### 5.3.2. Caseinolytic Peptidase b Protein Homolog (CLPB)

In a genome-wide CRISPR/Csa9 screen project aimed to identify synthetic vulnerabilities to BCL-2 inhibition in AML, sgRNAs targeting CLPB were found to sensitize AML blasts to venetoclax [36]. This study showed that CLPB, a mitochondrial chaperone protein, interacts with several antiapoptotic proteins in mitochondria and maintains the mitochondrial cristae structure. Depletion of CLPB induces the mitochondrial stress response and renders the AML more susceptible to venetoclax-induced apoptosis. Finally, the authors demonstrated that targeting CLPB overcomes p53-mediated venetoclax resistance and sensitizes AML cells to venetoclax and venetoclax/azacytidine treatments.

### 5.4. BET Inhibitors

BET proteins consist of BRD1/2/3/4 and bromodomain testis-specific protein (BRDT). This protein family contains the signature bromodomains that recognize histone acetylation and is involved in transcriptional initiation, elongation, and cell cycle regulation [128,129]. Since the first description of the BET inhibitors JQ1 [64] and I-BET [66] in 2010, many more BET targeting compounds have been developed and are currently in various stages of clinical trials [65]. In addition to the single-agent BET targeting that primarily inhibits the MYC expression [17], a combination of BET inhibitors with a second agent, such as the CDK9 inhibitor alvocidib, demonstrates a strong synergistic effect on suppressing AML [130]. Here, we summarized two recent studies that utilize the RNAi/CRISPR genetic screen approach to identify pathways mediating the therapeutic index of the BET inhibitors.

#### 5.4.1. Polycomb Repressive Complex 2 (PRC2)

PRC2, an H3K27 methyltransferase complex (Figure 2h), consists of several core subunits, including suppressor of zeste 12 protein homolog (SUZ12), embryonic ectoderm development (EED), and enhancer of zeste 1 and 2 (EZH1/EZH2) [131]. To identify epigenetic factors mediating sensitivity to BET inhibition, an shRNA library targeting 626 chromatin regulators (2917 shRNAs) was screened in a mouse MLL-r AML cell model treated with JQ1 [37,64]. This screen revealed that suppression of the PRC2 components (including SUZ12, EED, and EZH2) promotes BET inhibitor resistance in AML. Mechanistically, destruction of PRC2 activity maintains the MYC expression during BET inhibition by assisting the WNT signaling components in activating the MYC enhancer. Results from this study imply the rewiring of transcriptional programs through epigenetic targeting could serve as a strategy in combating BET inhibitor-resistant AML.

#### 5.4.2. Lysine-Specific Histone Demethylase 1A (KDM1A)

KDM1A, also known as LSD1, is a corepressor that mediates demethylation of H3K4 mono- and di-methylation (Figure 2i) [132]. KDM1A was found in a CRISPR/Cas9 screen (~1200 sgRNA targeting 693 epigenetic domains) that, when depleted, can induce the differentiation of I-BET-resistant AML blasts [38]. Mechanistically, targeting KDM1A re-sensitizes the resistant AML cells to I-BET treatment via facilitating the enhancer remodeling in the I-BET regulated genes. Interestingly, a recent study further investigated the modulators of KDM1A targeted therapy in AML using a genome-wide CRISPR library screen, and it revealed that combinational targeting of the mTORC1 pathway synergizes with the KDM1A inhibition to induce AML cell differentiation [133]. These studies highlight the utility of high-throughput genetic screens in discovering novel drug action/resistance mechanisms in AML therapy.

## 6. Conclusions and Perspectives

The application of large-scale sequencing efforts has uncovered the highly complicate genetic landscapes in AML patients [134]; however, a complete genetic and epigenetic dependency map of AML has yet to be elucidated. The development of sequence-specific gene modulation tools such as RNAi and CRISPR/Cas9, which enable high-throughput functional genetic screens when coupled with next-generation sequencing, represents an effective strategy to characterize the functional role of genetic changes in human diseases [135]. In this review, we summarized the genetic dependencies of AML identified through the RNAi and CRISPR screens. The AML-related genes identified via these screens significantly resembled cellular pathways mediating epigenetics, signaling transduction, transcriptional regulation, and energy metabolism. Interestingly, the effector genes targeting overlapping cellular pathways (e.g., KAT2A and SIRT1 can mediate acetylation and deacetylation of H3K9) could regulate AML through divergent down-stream targets, elaborating perplexing networks within the AML cells that fine-tune the leukemogenesis and therapeutic response. The success of high-throughput genetic screens in AML and other diseases also inspired the development of next-generation genetic screen tools such as the CRISPR interference (CRISPRi) and CRISPR activation (CRISPRa) systems [136,137] for more diverse gene functional editing. Besides identifying genes involved in proliferation/survival, novel genetic screen approaches such as combining RNAi/CRISPR libraries with cellular reporter/marker-sorting [138,139,140] will enable the discovery of mechanisms mediating specific signaling pathways or cellular processes such as AML cell differentiation. We foresee these innovative strategies will continue to explore the pathogenesis of AML and provide novel therapeutic options against this genetically heterogeneous disease.

## Figures and Tables

**Table 1:** Summary of AML-related genes identified via high-throughput genetic screens.

		Gene Identified	Type of Screen	Gene #	Construct #	Report Year	Ref #
**Epigenetic Regulators**	*Histone Writers*	MOF SETDB1 KAT2A HBO1	shRNA CRISPR/Cas9 CRISPR/Cas9 shRNA	468 ~350 18,010 270	2252 ~15–25 per gene 90,709 1922	2017 2017 2016 2020	[13] [14] [15] [16]
	*Histone Readers*	BRD4 ENL	shRNA CRISPR/Cas9	243 18,080	1094 64,751	2011 2017	[17] [18]
	*Histone Erasers*	JMJD1C SIRT1	shRNA shRNA	160 16,924	752 92,425	2016 2015	[19] [20]
**Kinase Pathways**		GSK3 ROCK1 PRL-3 CKMT1 LKB1	shRNA shRNA shRNA shRNA CRISPR/Cas9	~1000 16,000 67 482	~5000 7709 80,000 361 6 sgRNAs per gene	2012 2015 2018 2017 2018	[21] [22] [23] [24] [25]
**Genes Expression Regulators**		ZEB2 ZFP64 RBM25 RBM39	shRNA CRISPR/Cas9 shRNA CRISPR/Cas9	11,194 1426 230 490	54,020 8658 613 2900	2017 2018 2019 2019	[26] [27] [28] [29]
**Therapeutic Response Modulators**	*Cytarabine*	WEE1 DCK	siRNA CRISPR/Cas9	572 18,080	2 siRNAs per gene 64,751	2012 2016	[30] [31]
	*FLT3 inhibitors*	SPRY3 ATM GLS	CRISPR/Cas9 shRNA CRISPR/Cas9	18,080 18,010	64,751 ~4–5 per genes 90,709	2017 2016 2018	[32] [33] [34]
	*Venetoclax*	TP53 CLPB	CRISPR/Cas9 CRISPR/Cas9	18,010 18,675	90,709 110,257	2019 2019	[35] [36]
	*BET inhibitors*	PRC2 KDM1A	shRNA CRISPR/Cas9	626 693	2917 ~1200	2015 2019	[37] [38]
